# Comparative validation of automated perfusion analysis software for ischemic penumbra estimation and EVT decision-making

**DOI:** 10.3389/fnins.2025.1660870

**Published:** 2025-11-03

**Authors:** Jonguk Kim, Jong-Hyeok Park, Dongmin Kim, Myungjae Lee, Joon-Tae Kim, Leonard Sunwoo, Cheolkyu Jung, Wi-Sun Ryu, Beom Joon Kim

**Affiliations:** ^1^Department of Neurology, Seoul National University Bundang Hospital, College of Medicine, Seoul National University, Seongnam, Republic of Korea; ^2^Artificial Intelligence Research Center, JLK Inc., Seoul, Republic of Korea; ^3^Department of Neurology, Chonnam National University Hospital, College of Medicine, Chonnam National University, Gwangju, Republic of Korea; ^4^Department of Radiology, Seoul National University Bundang Hospital, College of Medicine, Seoul National University, Seongnam, Republic of Korea

**Keywords:** acute ischemic stroke, perfusion-weighted imaging, diffusion-weighted imaging, automated software, magnetic resonance imaging, endovascular thrombectomy

## Abstract

**Background:**

While computed tomography perfusion is widely used in acute stroke imaging, magnetic resonance perfusion-weighted imaging (PWI) offers superior spatial resolution and tissue specificity, particularly when combined with diffusion-weighted imaging (DWI). However, no prior study has systematically compared automated PWI analysis platforms. This study aims to evaluate the performance of a newly developed software (JLK PWI) against the established RAPID platform in terms of volumetric agreement and clinical decision concordance.

**Methods:**

This retrospective multicenter study included 299 patients with acute ischemic stroke who underwent PWI within 24 h of symptom onset. Volumetric agreement between RAPID and JLK PWI was assessed using concordance correlation coefficients (CCC), Bland–Altman plots, and Pearson correlations. Agreement in endovascular therapy (EVT) eligibility was evaluated using Cohen’s kappa based on DAWN and DEFUSE-3 criteria.

**Results:**

The mean age was 70.9 years, 55.9% were male, and the median NIHSS score was 11 (IQR 5–17). The median time from the last known well to PWI was 6.0 h. JLK PWI showed excellent agreement with RAPID for ischemic core (CCC = 0.87; *p* < 0.001) and hypoperfused volume (CCC = 0.88; *p* < 0.001). EVT eligibility classifications based on DAWN criteria showed very high concordance across subgroups (*κ* = 0.80–0.90), and substantial agreement was observed using DEFUSE-3 criteria (*κ* = 0.76).

**Conclusion:**

JLK PWI demonstrates high technical and clinical concordance with RAPID, supporting its use as a reliable alternative for MRI-based perfusion analysis in acute stroke care.

## Introduction

The advent of automated perfusion imaging analysis has significantly improved the triage of patients with acute ischemic stroke, particularly by extending the treatment window for endovascular therapy (Albers et al., 2018; Nogueira et al., 2018). Computed tomography perfusion (CTP) has become the predominant modality in emergency settings due to its rapid acquisition and broad accessibility (Kim et al., 2024b). As a result, many studies have compared CTP-based software platforms in terms of infarct core estimation, perfusion mismatch, and outcome prediction (Xiong et al., 2019; Suomalainen et al., 2022; Kim et al., 2024a).

In contrast, magnetic resonance perfusion-weighted imaging (PWI) has received less attention in the context of automated analysis. Previous studies have compared commercial PWI platforms to manual reference or reported differences between platforms (Galinovic et al., 2012; Chatterjee et al., 2015; Deutschmann et al., 2021; Xiong et al., 2022; Teichmann et al., 2025). However, these studies were generally limited by modest sample sizes, single-center designs, or the absence of direct comparative evaluations with RAPID across diverse stroke populations and clinical decision-making frameworks. This gap has hindered efforts to standardize MRI-based stroke workflows, despite their growing clinical applications.

PWI offers several technical advantages over CTP. It provides higher spatial resolution, is free from beam-hardening artifacts, and is less susceptible to contrast timing errors (Konstas et al., 2009; Liu et al., 2024). These features improve image quality, particularly in challenging regions such as the posterior fossa or in patients with small vessel disease. Additionally, when paired with DWI, PWI enables more accurate delineation of infarct core and penumbra (Kane et al., 2007), and avoids the risk of ionizing radiation exposure (Cohnen et al., 2006), making it suitable for selected patient populations and research contexts.

Recent clinical trials (Mohammaden et al., 2024; Goyal et al., 2025; Psychogios et al., 2025) targeting medium vessel occlusion (MeVO) have underscored the need for more refined imaging biomarkers to better identify patients who may benefit from treatment (Ospel et al., 2024; Salim et al., 2024; Cai et al., 2025). The combined spatial precision and tissue specificity of PWI-DWI may enhance patient stratification and inform more personalized treatment strategies.

In this study, we introduce a newly developed PWI analysis platform (JLK PWI, JLK Inc., Republic of Korea) and compare its performance with that of a widely used commercial software (RAPID, RAPID AI, CA, USA). We evaluate inter-platform agreement in volumetric parameters, including ischemic core, hypoperfused area, and mismatch volume, as well as in treatment eligibility based on DAWN and DEFUSE-3 trial criteria (Albers et al., 2018; Nogueira et al., 2018). This study aims to evaluate the clinical viability of JLK PWI as a robust alternative for MRI-based stroke assessment.

## Methods

### Study design and study population

This retrospective multicenter study included patients with acute ischemic stroke who underwent PWI within 24 h of symptom onset at two tertiary hospitals in Korea. A total of 216 patients from Seoul National University Bundang Hospital who underwent both PWI and endovascular thrombectomy between January 2019 and April 2024, and 102 patients from Chonnam National University Hospital who underwent PWI within 24 h of symptom onset with or without endovascular thrombectomy (EVT) between January 2015 and December 2015, were initially screened. After pooling the datasets, 318 patients met the inclusion criteria. Of these, patients were excluded due to abnormal arterial input function (*n* = 6), severe motion artifacts (*n* = 2), or inadequate images (*n* = 11). Consequently, 299 patients were included in the final analysis. The study protocol was approved by the institutional review board of Seoul National University Bundang Hospital [IRB# B-1710-429-102], and written informed consent was obtained from all patients or their legal representatives.

### Clinical data collection

Using a standardized protocol (Kim et al., 2014), we prospectively collected demographic data, vascular risk factors (hypertension, diabetes mellitus, hyperlipidemia, coronary artery disease, atrial fibrillation, and smoking history), prior medication use, pre-stroke functional status, and index stroke characteristics, such as initial stroke severity (NIH Stroke Scale, NIHSS) and subtypes. Stroke subtypes were determined by an experienced vascular neurologist, using a validated MRI-based classification system built on the TOAST (Trial of ORG 10172 in Acute Stroke Treatment) criteria (Ko et al., 2014).

### Imaging and image reconstruction

All perfusion MRI scans were performed on either 3.0 T (62.3%) or 1.5 T (37.7%) scanners. Regarding the vendors, 34.1% of scans were conducted using GE systems, 60.2% using Philips systems, and 5.7% using Siemens systems, all equipped with an 8-channel head coil. Dynamic susceptibility contrast-enhanced perfusion imaging was performed using a gradient-echo echo-planar imaging (GE-EPI) sequence. The imaging parameters were as follows: repetition time (TR) = 1,000–1,500 ms (6.3%), 1,500–2,000 ms (66.7%), or 2,000–2,500 ms (27.0%); echo time (TE) = 30–40 ms (1.0%), 40–50 ms (91.8%), or 60–70 ms (7.2%); field of view (FOV) = 210 × 210 mm^2^ (5.7%), or 230 × 230 mm^2^ (94.3%); and slice thickness of 5 mm with no interslice gap, covering the entire supratentorial brain with 17–25 slices. Images were reconstructed and exported in DICOM format for subsequent post-processing and quantitative perfusion analysis. To minimize inter-scanner variability, all datasets underwent standardized preprocessing and normalization prior to PWI mapping. All image analyses were done in the central image laboratory operated by Seoul National University Bundang Hospital.

### Automated PWI analysis

For infarct core estimation, RAPID employed the default threshold of ADC < 620 × 10^−6^ mm^2^/s. JLK PWI utilized a deep learning–based infarct segmentation algorithm applied to the b1000 DWI images, which was developed and validated in previous studies using large manually segmented datasets (Ryu et al., 2023, 2024, 2025).

**Figure 1:**
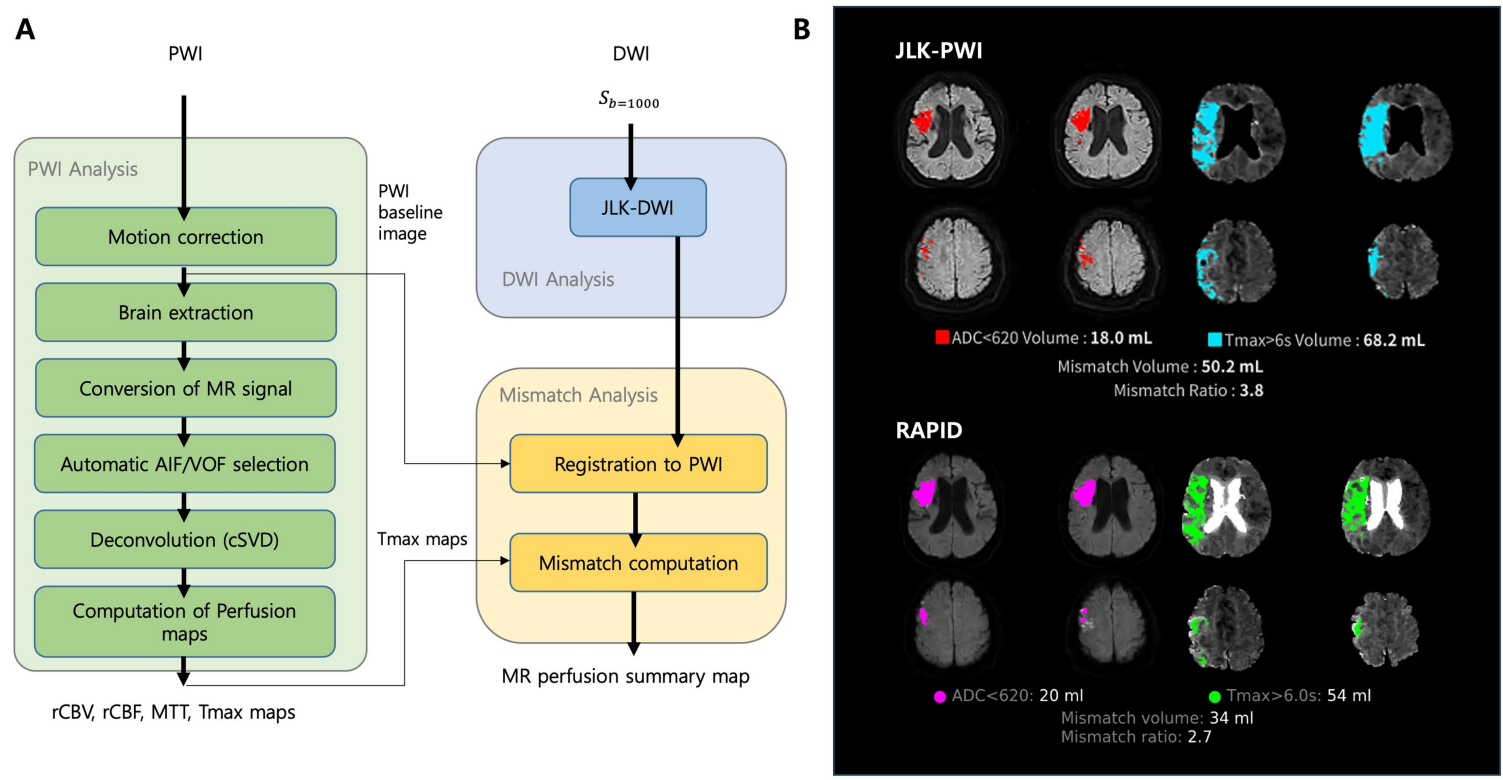
Two-panel figure showing MR perfusion workflow and example outputs. Panel **A** shows schematic pipeline for perfusion processing. Panel **B** represents a representative patient showing co-registered diffusion and perfusion maps with color overlays; ischemic core and hypoperfused tissue are quantified (mL) and mismatch ratio is displayed.

As illustrated in Figure 1A, JLK PWI performs automated preprocessing and perfusion parameter calculations through a multi-step pipeline. The workflow includes motion correction to acquisition artifacts, brain extraction by skull stripping and vessel masking, and conversion of MR signal. The software automatically selects the arterial input function and venous output function, followed by block-circulant single value deconvolution and calculation of quantitative perfusion maps, including CBF, CBV, MTT, and Tmax.

The infarct core from JLK-DWI is automatically co-registered to the perfusion maps, allowing mismatch computation between diffusion and perfusion lesions. The hypoperfused region was delineated using the threshold of Tmax >6 s. All segmentations and resulting images were visually inspected to ensure technical adequacy before inclusion in the analysis. Figure 1B presents a representative case comparing infarct core and hypoperfusion segmentation between JLK PWI and RAPID.

### Statistical analysis

Descriptive statistics were used to summarize baseline characteristics. Continuous variables were reported as means with standard deviations (SD) or medians with interquartile ranges (IQR), depending on data distribution. Categorical variables were presented as counts with percentages. Agreement between the two platforms in perfusion parameter measurements (ischemic core volume, hypoperfused volume, and mismatch volume) was assessed using concordance correlation coefficients (CCC), Pearson correlation coefficients, and Bland–Altman plots. The magnitude of agreement was classified as: poor (0.0–0.2), fair (0.21–0.40), moderate (0.41–0.60), substantial (0.61–0.80), and excellent (0.81–1.0) (Landis and Koch, 1977).

For EVT eligibility, classification agreement between the RAPID and JLK software was evaluated using Cohen’s kappa coefficient, applied separately for each subgroup defined by the DAWN and DEFUSE-3 trial criteria. The DAWN classification stratified eligible infarct volume based on age and NIHSS into three prespecified categories, while the DEFUSE-3 classification used a mismatch ratio ≥1.8, an infarct core volume <70 mL and an absolute volume of penumbra ≥15 mL. Cases with discordant EVT eligibility classifications were additionally analyzed descriptively.

Subgroup analyses were conducted for patients with anterior circulation large vessel occlusion (including internal carotid artery, middle cerebral artery M1-M2 branches, and anterior cerebral artery) and those with basilar artery occlusion. In each subgroup, agreement metrics and outcome prediction models were separately generated to evaluate software performance across stroke types. Additional analyses stratified by MRI vendor and field strength were conducted to assess the consistency of agreement across acquisition settings.

All statistical analyses were performed using STATA version 16.0 (StataCorp LLC, College Station, TX) and R version 4.2.3 (R Foundation for Statistical Computing, Vienna, Austria). A two-sided *p*-value < 0.05 was considered statistically significant.

### Subject characteristics

For 299 subjects included, the mean age was 70.9 years (SD 11.6), and 55.9% were male. The median NIHSS score on admission was 11 (IQR: 5–17). The most common stroke subtype was cardioembolism (45.2%), followed by large artery atherosclerosis (29.1%) and undetermined etiology (13.0%). Intravenous thrombolysis was administered in 157 patients (52.5%).

Regarding occlusion sites, 208 (69.6%) subjects had anterior circulation large vessel occlusion, and 31 had basilar artery occlusion (10.4%). Meanwhile, 60 (20.1%) subjects had no large vessel occlusion on MRI. The median time from the last known well to PWI was 360 min (IQR: 216–750) min, and the median time from PWI to groin puncture was 55.5 min (IQR: 40.8–82.3).

## Results

### Concordance of ischemic core, hypoperfused, and mismatch volumes

**Figure 2:**
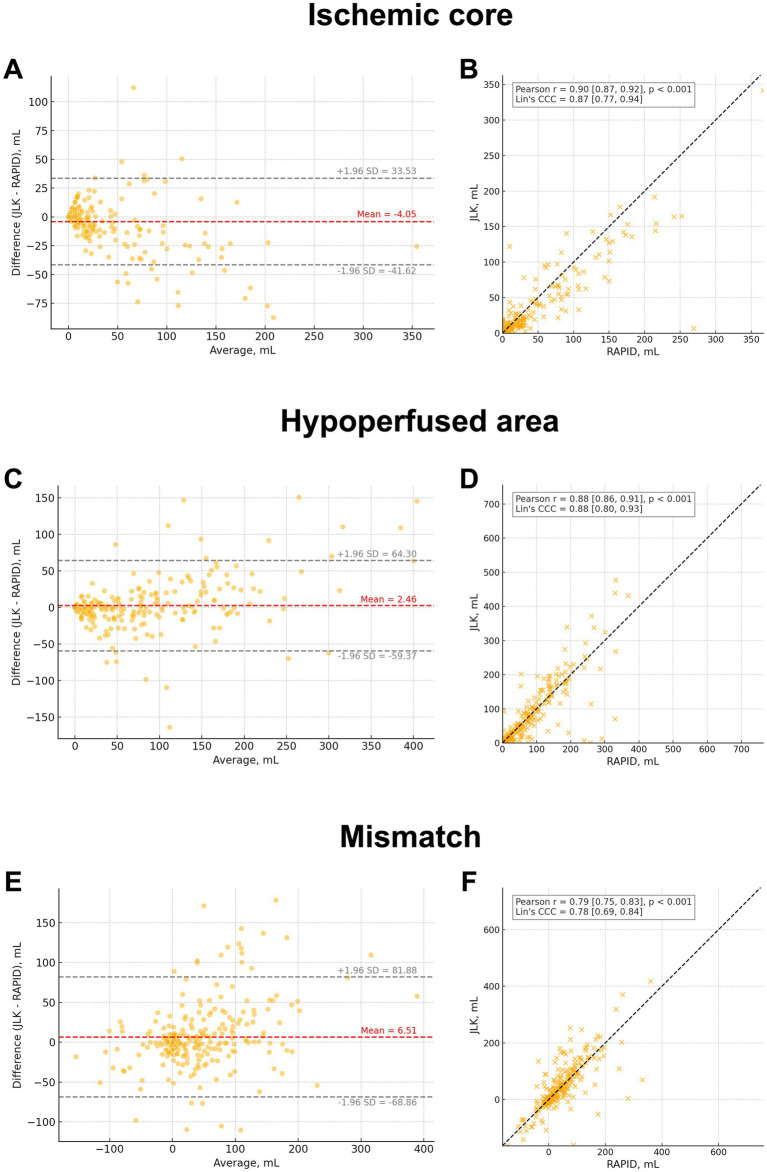
Agreement between JLK and RAPID perfusion outputs. Bland-Altman plots **(A, C, E)** show differences versus means for ischemic core, hypoperfused volume, and mismatch; central mean line and ±1.96 SD limits are drawn. Scatter plots **(B, D, F)** show correlations with linear fits; Pearson’s r and Lin’s concordance correlation coefficient (CCC) are reported. The panels summarize bias, limits of agreement, and strength of association across metrics, demonstrating high concordance for core and clinically meaningful agreement for hypoperfusion and mismatch.

Ischemic core volumes showed high agreement between RAPID and JLK PWI, with CCC = 0.87 (95% CI, 0.77–0.94; Figure 2B). The Bland–Altman plot showed a mean difference of −4.05 mL and limits of agreement ranging from −41.62 to 33.53 mL (Figure 2A). Similarly, hypoperfused volumes showed high agreement (CCC = 0.88 [95% CI, 0.80–0.93]; Figure 2D). The mean difference was 2.46 mL, with limits of agreement from −59.37 to 64.30 mL (Figure 2C). Mismatch volumes demonstrated substantial agreement (CCC = 0.78 [95% CI, 0.69–0.84]; Figure 2F), with a mean difference of 6.51 mL and limits of agreement from −68.86 to 81.88 mL (Figure 2E). Overall concordance was good, although relatively large volumetric discrepancies were observed in some subjects, as reflected in the wide limits of agreement (see Table 1).

**Table 1:** Baseline characteristics of the study population.

Variables	Values (*N* = 299)
Age, year	70.9 ± 11.6
Male	167 (55.9%)
Initial NIHSS score, IQR	11 [5–17]
Pre-stroke mRS ≤ 2	244 (81.6%)
Hypertension	197 (65.9%)
Diabetes	90 (30.1%)
Hyperlipidemia	118 (39.5%)
Smoking	79 (26.4%)
Atrial fibrillation	131 (43.8%)
Stroke subtype	
Large artery atherosclerosis	87 (29.1%)
Cardioembolism	135 (45.2%)
Small vessel occlusion	25 (8.4%)
Undetermined	39 (13.0%)
Other determined	13 (4.4%)
MR manufacturer	
GE	102 (34.1%)
Siemens	17 (5.7%)
Philips	180 (60.2%)
Magnetic field strength, T	
1.5	117 (39.1%)
3	182 (60.9%)
Intravenous thrombolysis	157 (52.5%)
Endovascular thrombectomy	214 (71.6%)
Occlusion site	
Anterior circulation large vessel occlusion^a^	208 (69.6%)
Basilar artery occlusion	31 (10.4%)
No large vessel occlusion	60 (20.1%)
Time indices	
Last known well to PWI, min	360 [216–750]
PWI to puncture, min (*n* = 214)^b^	55.5 [40.8–82.3]

Data were presented as mean ± standard deviation, median [interquartile range], or number (percentage).

NIHSS, National Institutes of Health Stroke Scale; mRS, modified Rankin Scale; PWI, perfusion-weighted image.

a Defined as occlusion of the intracranial internal carotid artery, middle cerebral artery M1–M2 branches, and anterior cerebral artery.

b Only in patients who have undergone EVT.

Subgroup analyses for patients with anterior circulation large vessel occlusion (Supplementary Figure 1) and basilar artery occlusion (Supplementary Figure 2) showed similar trends in agreement across core, hypoperfusion, and mismatch volumes. In the basilar artery occlusion group (*n* = 31), ischemic core volumes demonstrated high agreement between RAPID and JLK PWI (CCC = 0.95 [95% CI, 0.88–0.97]), whereas hypoperfusion volumes showed moderate agreement with CCC = 0.55 (95% CI, 0.31–0.81).

Additional subgroup analyses by different field strengths and MRI vendors demonstrated consistently high concordance across scanner types, with comparable CCC values for ischemic core, hypoperfused, and mismatch volumes (Supplementary Figures 3–7).

### Concordance of EVT eligibility based on DAWN and DEFUSE-3 criteria

**Figure 3:**
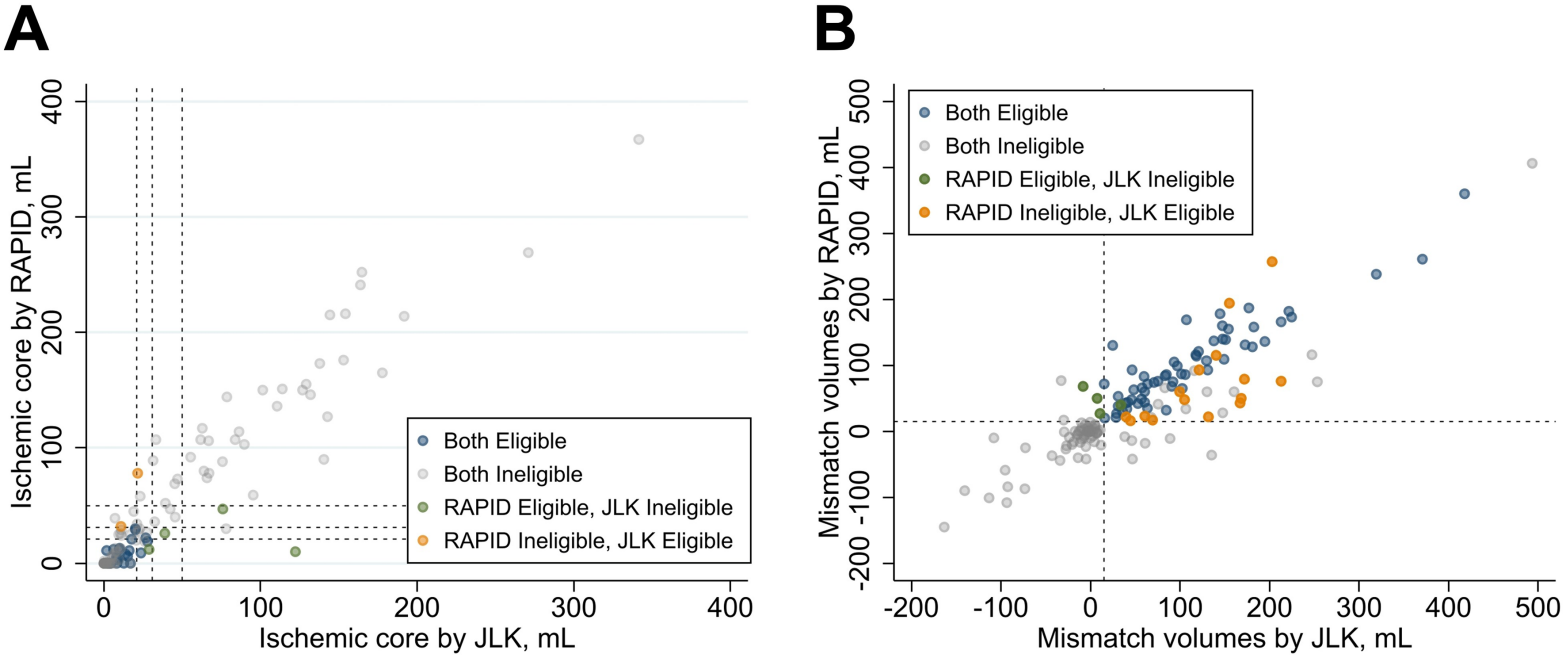
Outcome of selection criteria using RAPID versus JLK. Panel **A** compares ischemic core volumes (mL); Panel **B** compares mismatch volumes (mL). Points are colored by eligibility: both eligible (blue), both ineligible (gray), RAPID-only eligible (green), and JLK-only eligible (orange).

To assess the concordance in determining eligibility for EVT, we applied the strict inclusion criteria from the DAWN and DEFUSE-3 trials to the relevant subgroups within our patient cohort. For the DAWN trial criteria, the analysis included 123 patients with an anterior circulation large vessel occlusion and an initial NIHSS score of 10 or higher. In this subgroup, the agreement between RAPID and JLK PWI was excellent (Cohen’s *κ* = 0.873; 95% CI, 0.773–0.973; Table 2, Figure 3A).

**Table 2:** Assessment of RAPID vs. JLK software in determining eligibility for endovascular thrombectomy based on DAWN and DEFUSE 3 trial criteria.

Trial criteria		RAPID	JLK PWI		Cohen’s kappa (95% CI)
			Not eligible	Eligible	
DAWN	All (*N* = 123)^a^	Not eligible	88	2	0.873 (0.773 to 0.973)
		Eligible	4	29	
	Age >80 and NIHSS score ≥10 (*n* = 37)	Not eligible	28	0	0.841 (0.620 to 1.000)
		Eligible	2	7	
	Age ≤80 and NIHSS score 10–19 (*n* = 76)	Not eligible	55	2	0.897 (0.779 to 1.000)
		Eligible	1	18	
	Age ≤80 and NIHSS score ≥20 (*n* = 10)	Not eligible	5	0	0.800 (0.357 to 1.000)
		Eligible	1	4	
DEFUSE 3 (*N* = 163)^b^		Not eligible	86	15	0.761 (0.660 to 0.862)
		Eligible	4	58	

a Patients with initial NIHSS of 10 or more and anterior circulation large vessel occlusion were included.

b Patients with initial NIHSS of 6 or more and anterior circulation large vessel occlusion were included.

For the DEFUSE-3 trial criteria, the analysis included 163 patients with an anterior circulation large vessel occlusion and an initial NIHSS score of 6 or higher. The agreement between the platforms was substantial (Cohen’s *κ* = 0.761; 95% CI, 0.660–0.862). Both platforms concordantly identified 58 patients as eligible and 86 as ineligible. There were 19 discordant cases, with 15 deemed eligible by JLK PWI only and 4 by RAPID only (Table 2, Figure 3B). A detailed breakdown of the discordant classifications for DEFUSE-3 is provided in Supplementary Table 1. For the 15 patients deemed eligible only by JLK PWI, the most common reason for ineligibility by RAPID was an ischemic core volume >70 mL (13 of 15 cases). For the four patients deemed eligible only by RAPID, the primary reasons for ineligibility by JLK PWI were a mismatch volume <15 mL (3 of 4 cases) and a mismatch ratio <1.8 (1 of 4 cases).

## Discussion

To our knowledge, this study is among the first to conduct a comprehensive validation of a newly developed MRI perfusion software (JLK PWI) against the established RAPID platform, using both volumetric and clinical decision-making metrics. Importantly, our analysis is not limited to core–hypoperfusion volume comparisons but also includes EVT triage concordance based on DAWN and DEFUSE-3 criteria, as well as volumetric concordance of infarct core estimation between JLK PWI and diffusion-restricted lesions defined by RAPID (ADC < 620). This multifaceted approach offers a pragmatic perspective for assessing real-world performance of automated perfusion software within acute stroke workflows.

A notable strength of our study lies in its inclusion of broad stroke population, encompassing both anterior and posterior circulation large vessel occlusion, and a wide spectrum of imaging time windows up to 24 h. Most prior validation studies have focused on CTP-derived perfusion maps or DWI-based core estimation alone (Austein et al., 2016; Suomalainen et al., 2022). By leveraging PWI-DWI integration in a clinical setting, we demonstrate that the JLK PWI achieves excellent volumetric agreement with RAPID (CCC = 0.87) and high agreement in EVT decision-making (Cohen’s *κ* up to 0.90 for DAWN). These results support the use of JLK PWI not only as a technical substitute, but also as a clinical decision-making tool (Neumann-Haefelin et al., 1999; Mishra et al., 2025).

Accurate, automated estimation of infarct core is critical for patient selection in reperfusion therapies, particularly in extended time windows and in settings where CTP is unavailable or unsuitable (Evans et al., 2018; Mishra et al., 2025). Our findings show that JLK PWI maintains high fidelity in infarct core and hypoperfusion volume estimation across diverse patient profiles. Notably, EVT eligibility classifications showed high concordance between the two platforms, with 95% agreement for DAWN (*κ* = 0.873) and 88% agreement for DEFUSE-3 (*κ* = 0.761). These findings suggest that JLK PWI can be effective in guideline-based treatment decisions.

It is noteworthy that the agreement rate for DAWN criteria is substantially higher than that for DEFUSE-3. A plausible explanation is that JLK PWI showed more consistent performance in estimating the infarct core on DWI, which is central to the clinical-DWI mismatch approach underlying DAWN. In contrast, DEFUSE-3 additionally incorporates hypoperfusion and mismatch volumes from PWI, where greater variability was observed between the two platforms. This variability may be related to differences in imaging characteristics. DWI provides higher spatial resolution and more reliable lesion delineation, whereas PWI is more sensitive to contrast timing and post-processing (Demeestere et al., 2020). Also, variation in hypoperfusion segmentation algorithms across platforms may contribute. These findings suggest that PWI-based mismatch measurements should be interpreted with caution when applied in clinical decision-making.

Furthermore, JLK PWI software demonstrated good agreement for ischemic core estimation and reasonable agreement for hypoperfusion volumes even in posterior circulation strokes, where perfusion analysis remains technically challenging (Pallesen et al., 2018). While the number of patients with basilar artery occlusion in our cohort was limited, these findings suggest its potential utility in future studies and clinical protocols involving basilar occlusions or MeVOs, which are increasingly recognized as important therapeutic targets despite the current lack of standardized imaging criteria (Cimflova et al., 2021; Alemseged et al., 2023).

Some limitations must be acknowledged. First, the generalizability of our findings may be limited by the retrospective design and the inclusion of two tertiary stroke centers. Second, MRI scans were acquired using both 3.0 T and 1.5 T systems from different vendors, and heterogeneity in acquisition parameters and contrast timing may influence software outputs. Third, we did not include specific imaging data such as infarct growth or collateral status (de Havenon et al., 2019), which could provide additional context for discrepancies in EVT decision classification.

Future studies should prospectively validate JLK PWI in broader clinical settings, including underrepresented stroke populations such as MeVOs and wake-up strokes. Integration of additional imaging biomarkers, such as collateral grading (Tetteh et al., 2023), or radiomic texture features (Li et al., 2024), may further enhance prediction models and assist in complex clinical decisions. In addition, longitudinal studies examining infarct evolution and clinical outcomes after EVT could provide insight into the long-term predictive validity of automated core estimation tools like JLK PWI.

## Conclusion

In conclusion, JLK PWI demonstrates excellent technical and clinical agreement with established commercial software, offering reliable infarct core estimation and high concordance in EVT decision-making. Its applicability across diverse stroke types and its foundation in deep learning–based analysis position it as a promising tool for real-time stroke triage. As imaging-based selection becomes increasingly nuanced, JLK PWI may play a critical role in improving access to individualized, time-sensitive reperfusion therapy.

## Data Availability

The original contributions presented in the study are included in the article/Supplementary material, further inquiries can be directed to the corresponding authors.

## References

[ref1] Albers G. W. Marks M. P. Kemp S. Christensen S. Tsai J. P. Ortega-Gutierrez S.. (2018). Thrombectomy for stroke at 6 to 16 hours with selection by perfusion imaging. N. Engl. J. Med. 378, 708–718. doi: 10.1056/nejmoa1713973, PMID: 29364767 PMC6590673

[ref2] Alemseged F. Nguyen T. N. Alverne F. M. Liu X. Schonewille W. J. Nogueira R. G. (2023). Endovascular therapy for basilar artery occlusion. Stroke 54, 1127–1137. doi: 10.1161/strokeaha.122.040807, PMID: 36722343

[ref3] Austein F. Riedel C. Kerby T. Meyne J. Binder A. Lindner T.. (2016). Comparison of perfusion CT software to predict the final infarct volume after thrombectomy. Stroke 47, 2311–2317. doi: 10.1161/strokeaha.116.013147, PMID: 27507864

[ref4] Cai L. Y. Hoseinyazdi M. Lakhani D. A. Salim H. Mei J. Dmytriw A. A.. (2025). Redefining ischemic core, penumbra, and target mismatch on perfusion imaging in acute anterior distal medium vessel occlusion. Stroke Vasc. Interv. Neurol. doi: 10.1161/svin.125.001900 PMC12697630 41608706

[ref5] Chatterjee N. R. Ansari S. A. Vakil P. Prabhakaran S. Carroll T. J. Hurley M. C. (2015). Automated analysis of perfusion weighted MRI using asymmetry in vascular territories. Magn. Reson. Imaging 33, 618–623. doi: 10.1016/j.mri.2015.01.009, PMID: 25601529 PMC4426216

[ref6] Cimflova P. McDonough R. Kappelhof M. Singh N. Kashani N. Ospel J. M.. (2021). Perceived limits of endovascular treatment for secondary medium-vessel-occlusion stroke. Am. J. Neuroradiol. 42, 2188–2193. doi: 10.3174/ajnr.a7327, PMID: 34711552 PMC8805763

[ref7] Cohnen M. Wittsack H.-J. Assadi S. Muskalla K. Ringelstein A. Poll L. W.. (2006). Radiation exposure of patients in comprehensive computed tomography of the head in acute stroke. AJNR Am. J. Neuroradiol. 27, 1741–1745. 16971627 PMC8139787

[ref8] de Havenon A. Mlynash M. Kim-Tenser M. A. Lansberg M. G. Leslie-Mazwi T. Christensen S.. (2019). Results from DEFUSE 3. Stroke 50, 632–638. doi: 10.1161/strokeaha.118.023407, PMID: 30726184 PMC6628906

[ref9] Demeestere J. Wouters A. Christensen S. Lemmens R. Lansberg M. G. (2020). Review of perfusion imaging in acute ischemic stroke. Stroke 51, 1017–1024. doi: 10.1161/strokeaha.119.028337, PMID: 32008460

[ref10] Deutschmann H. Hinteregger N. Wießpeiner U. Kneihsl M. Fandler-Höfler S. Michenthaler M.. (2021). Automated MRI perfusion-diffusion mismatch estimation may be significantly different in individual patients when using different software packages. Eur. Radiol. 31, 658–665. doi: 10.1007/s00330-020-07150-8, PMID: 32822053 PMC7813720

[ref11] Evans J. W. Graham B. R. Pordeli P. Al-Ajlan F. S. Willinsky R. Montanera W. J.. (2018). Time for a time window extension: insights from late presenters in the ESCAPE trial. Am. J. Neuroradiol. 39, 102–106. doi: 10.3174/ajnr.a5462, PMID: 29191873 PMC7410688

[ref12] Galinovic I. Ostwaldt A.-C. Soemmer C. Bros H. Hotter B. Brunecker P.. (2012). Automated vs manual delineations of regions of interest – a comparison in commercially available perfusion MRI software. BMC Med. Imaging 12:16. doi: 10.1186/1471-2342-12-16, PMID: 22809148 PMC3423015

[ref13] Goyal M. Ospel J. M. Ganesh A. Dowlatshahi D. Volders D. Möhlenbruch M. A.. (2025). Endovascular treatment of stroke due to medium-vessel occlusion. N. Engl. J. Med. 392, 1385–1395. doi: 10.1056/nejmoa2411668, PMID: 39908448

[ref14] Kane I. Sandercock P. Wardlaw J. (2007). Magnetic resonance perfusion diffusion mismatch and thrombolysis in acute ischaemic stroke: a systematic review of the evidence to date. J. Neurol. Neurosurg. Psychiatry 78, 485–491. doi: 10.1136/jnnp.2006.100347, PMID: 17056631 PMC2117840

[ref15] Kim N. Ha S. Y. Park G.-H. Park J.-H. Kim D. Sunwoo L.. (2024a). Comparison of two automated CT perfusion software packages in patients with ischemic stroke presenting within 24 h of onset. Front. Neurosci. 18:1398889. doi: 10.3389/fnins.2024.1398889, PMID: 38868398 PMC11168493

[ref16] Kim B. J. Park J.-M. Kang K. Lee S. J. Ko Y. Kim J. G.. (2014). Case characteristics, hyperacute treatment, and outcome information from the clinical research Center for Stroke-Fifth Division registry in South Korea. J. Stroke 17, 38–53. doi: 10.5853/jos.2015.17.1.38 PMC4325643 25692106

[ref17] Kim N. Ryu W. Ha S. Y. Kim J. Y. Kang J. Baik S. H.. (2024b). Optimal cerebral blood flow thresholds for ischemic core estimation using computed tomography perfusion and diffusion-weighted imaging. Ann. Neurol. 97, 919–929. doi: 10.1002/ana.27169, PMID: 39723650

[ref18] Ko Y. Lee S. Chung J.-W. Han M.-K. Park J.-M. Kang K.. (2014). MRI-based algorithm for acute ischemic stroke subtype classification. J Stroke 16, 161–172. doi: 10.5853/jos.2014.16.3.161, PMID: 25328874 PMC4200592

[ref19] Konstas A. A. Goldmakher G. V. Lee T.-Y. Lev M. H. (2009). Theoretic basis and technical implementations of CT perfusion in acute ischemic stroke, part 1: theoretic basis. Am. J. Neuroradiol. 30, 662–668. doi: 10.3174/ajnr.a1487, PMID: 19270105 PMC7051780

[ref20] Landis J. R. Koch G. G. (1977). The measurement of observer agreement for categorical data. Biometrics 33, 159–174. doi: 10.2307/2529310, PMID: 843571

[ref21] Li M. Jiang J. Hongmei G. Su H. Jingli W. Hu C. (2024). CT-based intra-thrombus and peri-thrombus radiomics for prediction of prognosis after endovascular thrombectomy: a retrospective study across two centers. Am. J. Neuroradiol. 46:ajnr.A8522. doi: 10.3174/ajnr.a8522 PMC11979854 39366763

[ref22] Liu M. Wen X. Li M. Huang Q. Jiang C. Jiang J.. (2024). Blind spots in brain imaging: a pictorial essay. Quant. Imaging Med. Surg. 15, 1021039–1023039. doi: 10.21037/qims-24-1270 PMC11744183 39839019

[ref23] Mishra N. K. Albers G. W. Christensen S. Marks M. Hamilton S. Straka M.. (2025). Comparison of magnetic resonance imaging mismatch criteria to select patients for endovascular stroke therapy. Stroke 45, 1369–1374. doi: 10.1161/strokeaha.114.004772 PMC4007191 24699054

[ref24] Mohammaden M. H. Viana L. S. Abdelhamid H. Olive-Gadea M. Rodrigo-Gisbert M. Requena M.. (2024). Endovascular versus medical management in distal medium vessel occlusion stroke: the DUSK study. Stroke 55, 1489–1497. doi: 10.1161/strokeaha.123.045228, PMID: 38787927

[ref25] Neumann-Haefelin T. Wittsack H.-J. Wenserski F. Siebler M. Seitz R. J. Mödder U.. (1999). Diffusion- and perfusion-weighted MRI. Stroke 30, 1591–1597. doi: 10.1161/01.str.30.8.1591, PMID: 10436106

[ref26] Nogueira R. G. Jadhav A. P. Haussen D. C. Bonafe A. Budzik R. F. Bhuva P.. (2018). Thrombectomy 6 to 24 hours after stroke with a mismatch between deficit and infarct. N. Engl. J. Med. 378, 11–21. doi: 10.1056/nejmoa1706442, PMID: 29129157

[ref27] Ospel J. M. Nguyen T. N. Jadhav A. P. Psychogios M.-N. Clarençon F. Yan B.. (2024). Endovascular treatment of medium vessel occlusion stroke. Stroke 55, 769–778. doi: 10.1161/strokeaha.123.036942, PMID: 38235587

[ref28] Pallesen L. -P. Lambrou D. Eskandari A. Barlinn J. Barlinn K. Reichmann H.. (2018). Perfusion computed tomography in posterior circulation stroke: predictors and prognostic implications of focal hypoperfusion. Eur. J. Neurol. 25, 725–731. doi: 10.1111/ene.13578 29350878

[ref29] Psychogios M. Brehm A. Ribo M. Rizzo F. Strbian D. Räty S.. (2025). Endovascular treatment for stroke due to occlusion of medium or distal vessels. N. Engl. J. Med. 392, 1374–1384. doi: 10.1056/nejmoa2408954, PMID: 39908430

[ref30] Ryu W.-S. Kang Y.-R. Noh Y.-G. Park J.-H. Kim D. Kim B. C.. (2023). Acute infarct segmentation on diffusion-weighted imaging using deep learning algorithm and RAPID MRI. J. Stroke 25, 425–429. doi: 10.5853/jos.2023.02145, PMID: 37813675 PMC10574298

[ref31] Ryu W.-S. Schellingerhout D. Lee H. Lee K.-J. Kim C. K. Kim B. J.. (2024). Deep learning-based automatic classification of ischemic stroke subtype using diffusion-weighted images. J. Stroke 26, 300–311. doi: 10.5853/jos.2024.00535, PMID: 38836277 PMC11164582

[ref32] Ryu W.-S. Schellingerhout D. Park J. Chung J. Jeong S.-W. Gwak D.-S.. (2025). Deep learning-based automatic segmentation of cerebral infarcts on diffusion MRI. Sci. Rep. 15:13214. doi: 10.1038/s41598-025-91032-w, PMID: 40240396 PMC12003832

[ref33] Salim H. A. Vagal V. Lakhani D. A. Mei J. Luna L. Aziz Y.. (2024). Association of pretreatment perfusion imaging parameters with 90-day excellent functional outcomes in anterior circulation distal medium vessel occlusion stroke. Am. J. Neuroradiol. 46:ajnr.A8584. doi: 10.3174/ajnr.a8584 PMC12091971 39547803

[ref34] Suomalainen O. P. Martinez-Majander N. Sibolt G. Bäcklund K. Järveläinen J. Korvenoja A.. (2022). Comparative analysis of core and perfusion lesion volumes between commercially available computed tomography perfusion software. Eur. Stroke J. 8, 259–267. doi: 10.1177/23969873221135915, PMID: 37021148 PMC10069177

[ref35] Teichmann L.-S. J. Khalil A. A. Villringer K. Fiebach J. B. Huwer S. Gibson E.. (2025). Evaluation of Siemens Healthineers’ StrokeSegApp for automated diffusion and perfusion lesion segmentation in patients with ischemic stroke. Front. Neurol. 16:1518477. doi: 10.3389/fneur.2025.1518477, PMID: 39926019 PMC11804811

[ref36] Tetteh G. Navarro F. Meier R. Kaesmacher J. Paetzold J. C. Kirschke J. S.. (2023). A deep learning approach to predict collateral flow in stroke patients using radiomic features from perfusion images. Front. Neurol. 14:1039693. doi: 10.3389/fneur.2023.1039693, PMID: 36895903 PMC9990868

[ref37] Xiong Y. Huang C. C. Fisher M. Hackney D. B. Bhadelia R. A. Selim M. H. (2019). Comparison of automated CT perfusion Softwares in evaluation of acute ischemic stroke. J. Stroke Cerebrovasc. Dis. 28:104392. doi: 10.1016/j.jstrokecerebrovasdis.2019.104392, PMID: 31562038

[ref38] Xiong Y. Luo Y. Wang M. Yang S.-T. Shi R. Ye W.. (2022). Evaluation of diffusion–perfusion mismatch in acute ischemic stroke with a new automated perfusion-weighted imaging software: a retrospective study. Neurol. Ther. 11, 1777–1788. doi: 10.1007/s40120-022-00409-w, PMID: 36201112 PMC9588132

