# Supplementary material for: Comparative validation of automated perfusion analysis software for ischemic penumbra estimation and EVT decision-making
Source: Front Neurosci. 2025 Nov 3;19:1660870. doi: 10.3389/fnins.2025.1660870 (PMC12620487; doi:10.3389/fnins.2025.1660870)

**Comparative Validation of Automated Perfusion Analysis Software for Ischemic Penumbra Estimation and EVT Decision-Making**

Supplementary Table 1. Reasons for Discordant EVT Eligibility Classification between RAPID and JLK PWI Using DEFUSE-3 Criteria.

Supplementary Figure 1. Concordance of Ischemic Core, Hypoperfused Area, and Mismatch Volumes Between Platforms in Patients with Anterior Circulation Large Vessel Occlusion.

Supplementary Figure 2. Concordance of Ischemic core, Hypoperfused Area, and Mismatch Volumes Between Platforms in patients with Basilar Artery Occlusion.

Supplementary Figure 3. Concordance of Ischemic core, Hypoperfused Area, and Mismatch Volumes Between Platforms in patients scanned on 1.5T MRI systems.

Supplementary Figure 4. Concordance of Ischemic core, Hypoperfused Area, and Mismatch Volumes Between Platforms in patients scanned on 3.0T MRI systems.

Supplementary Figure 5. Concordance of Ischemic core, Hypoperfused Area, and Mismatch Volumes Between Platforms in patients scanned using GE MRI systems.

Supplementary Figure 6. Concordance of Ischemic core, Hypoperfused Area, and Mismatch Volumes Between Platforms in patients scanned using Philips MRI systems.

Supplementary Figure 7. Concordance of Ischemic core, Hypoperfused Area, and Mismatch Volumes Between Platforms in patients scanned using Siemens MRI systems.

Supplementary Table 1. Reasons for Discordant EVT Eligibility Classification between RAPID and JLK PWI Using DEFUSE-3 Criteria.

| Discordant Classification | Reason for Ineligibility by the Other Platform | Number of Patients |
| --- | --- | --- |
| Eligible by JLK PWI only | Ischemic Core Volume > 70 mL | 13 |
|  | Mismatch Ratio < 1.8 | 2 |
|  | Mismatch Volume < 15 mL | 0 |
| Eligible by RAPID only | Ischemic Core Volume > 70 mL | 0 |
|  | Mismatch Ratio < 1.8 | 1 |
|  | Mismatch Volume < 15 mL | 3 |

**Supplementary Figure 1. Concordance of Ischemic Core, Hypoperfused Area, and Mismatch Volumes Between Platforms in Patients with Anterior Circulation Large Vessel Occlusion.** (A) Bland–Altman plot and (B) scatter plot for ischemic core volumes. (C) Bland–Altman plot and (D) scatter plot for hypoperfused volumes. (E) Bland–Altman plot and (F) scatter plot for mismatch volumes. Red dotted lines indicate the mean difference, and gray dotted lines represent the limits of agreement between the two platforms.


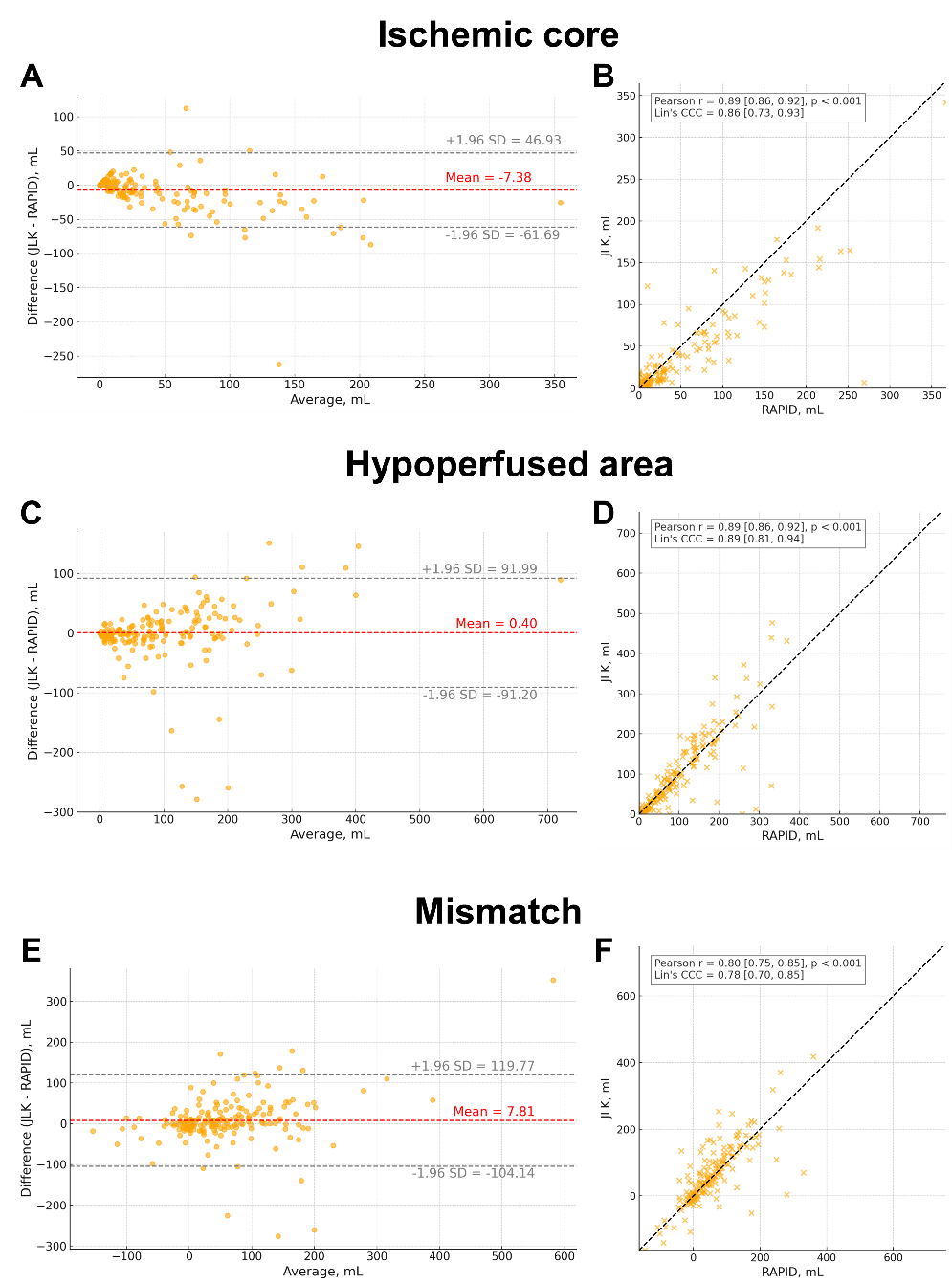


**Supplementary Figure 2. Concordance of Ischemic core, Hypoperfused Area, and Mismatch Volumes Between Platforms in patients with Basilar Artery Occlusion.** (A) Bland–Altman plot and (B) scatter plot for ischemic core volumes. (C) Bland–Altman plot and (D) scatter plot for hypoperfused volumes. (E) Bland–Altman plot and (F) scatter plot for mismatch volumes. Red dotted lines indicate the mean difference, and gray dotted lines represent the limits of agreement between the two platforms.


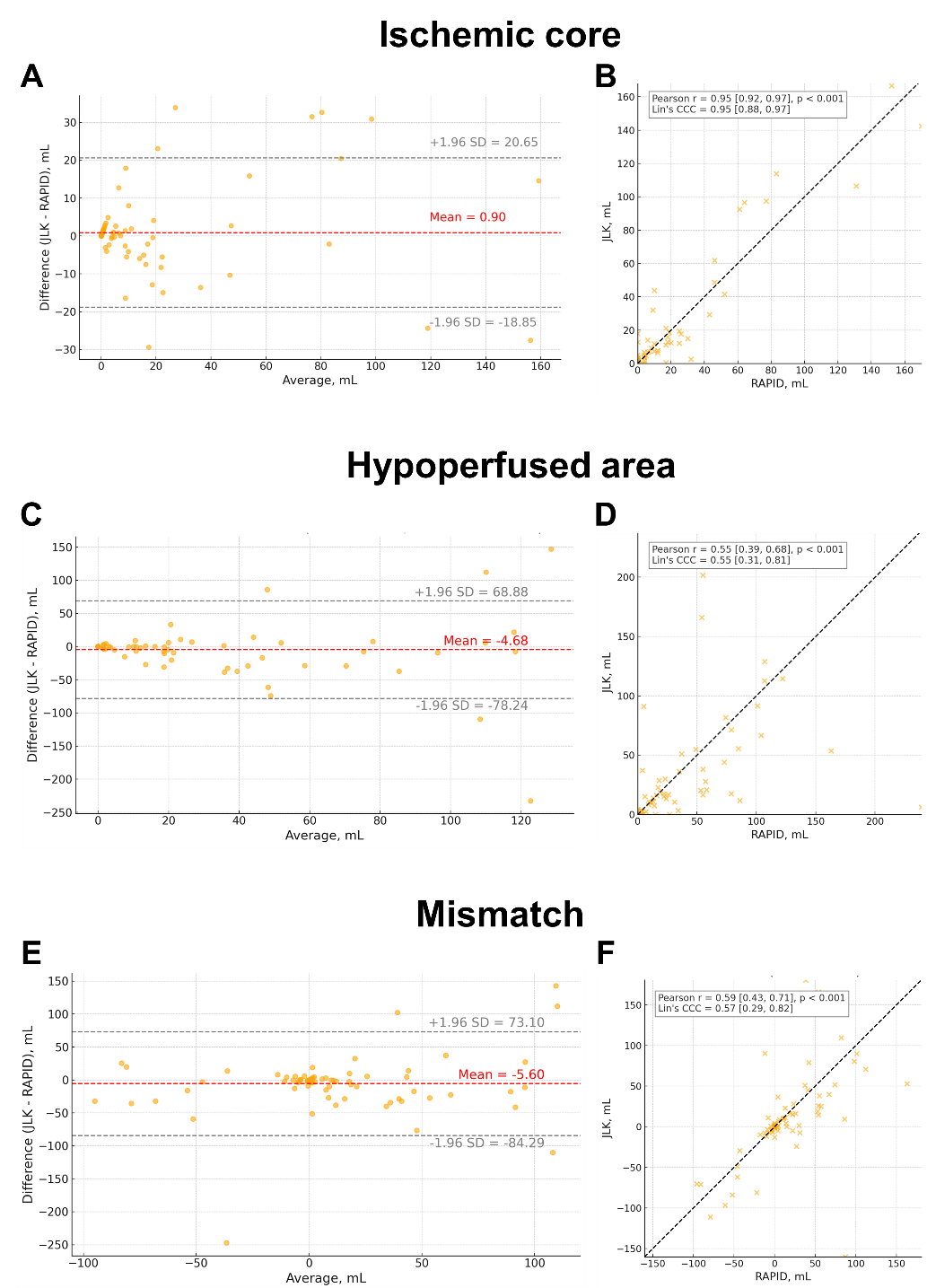


**Supplementary Figure 3. Concordance of Ischemic core, Hypoperfused Area, and Mismatch Volumes Between Platforms in patients scanned on 1.5T MRI systems.**

**
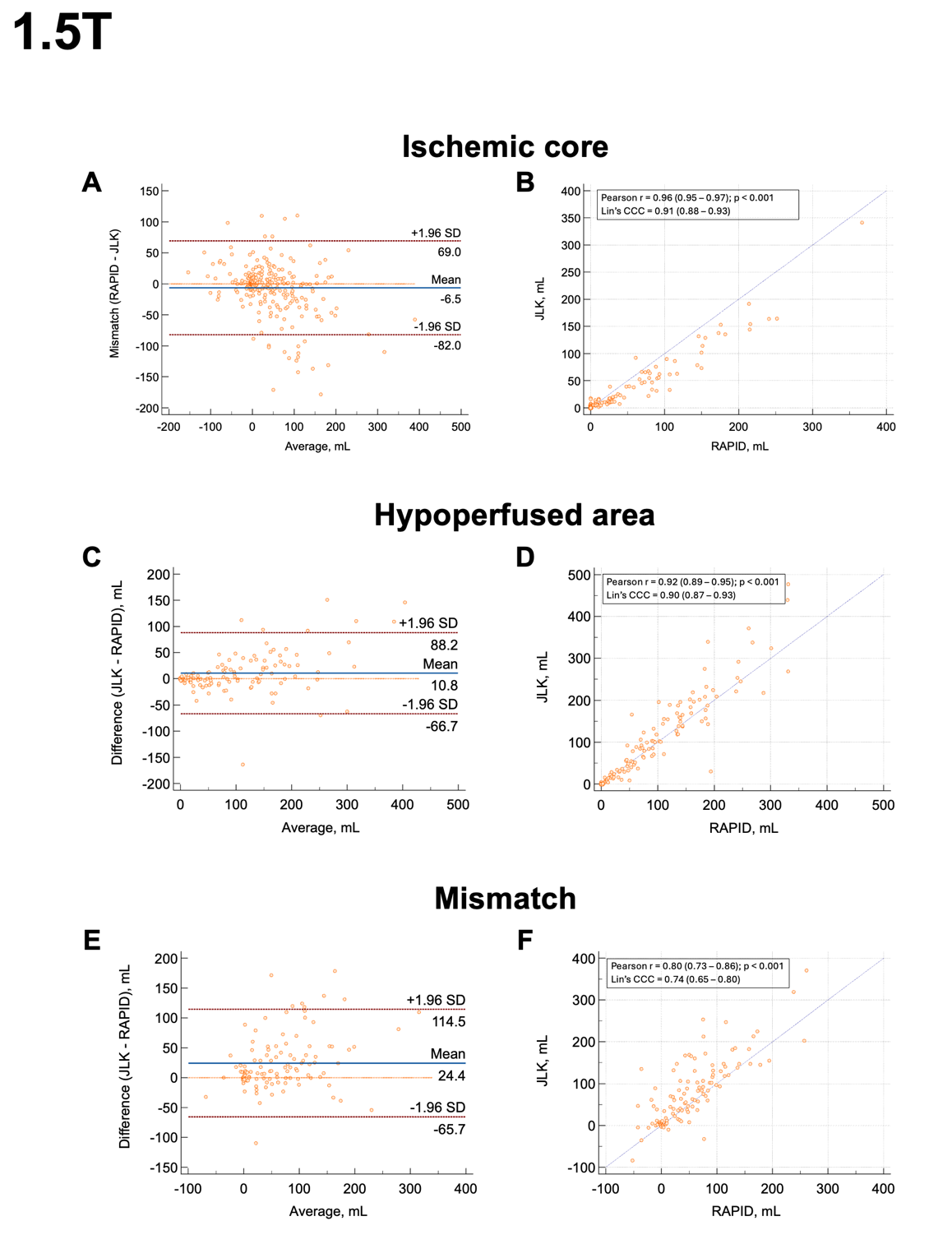
**

**Supplementary Figure 4. Concordance of Ischemic core, Hypoperfused Area, and Mismatch Volumes Between Platforms in patients scanned on 3.0T MRI systems.**

**
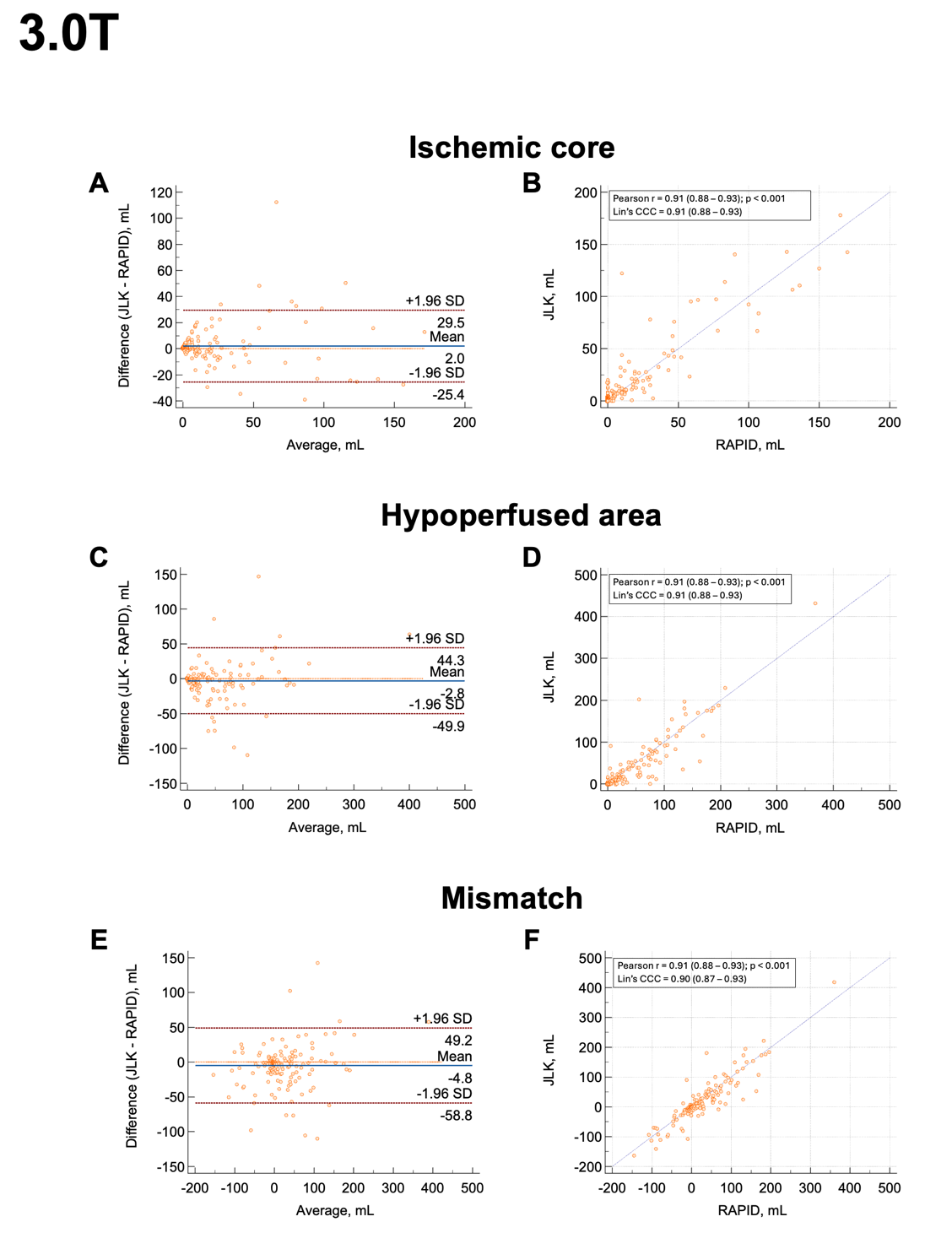
**

**Supplementary Figure 5. Concordance of Ischemic core, Hypoperfused Area, and Mismatch Volumes Between Platforms in patients scanned using GE MRI systems.**

**
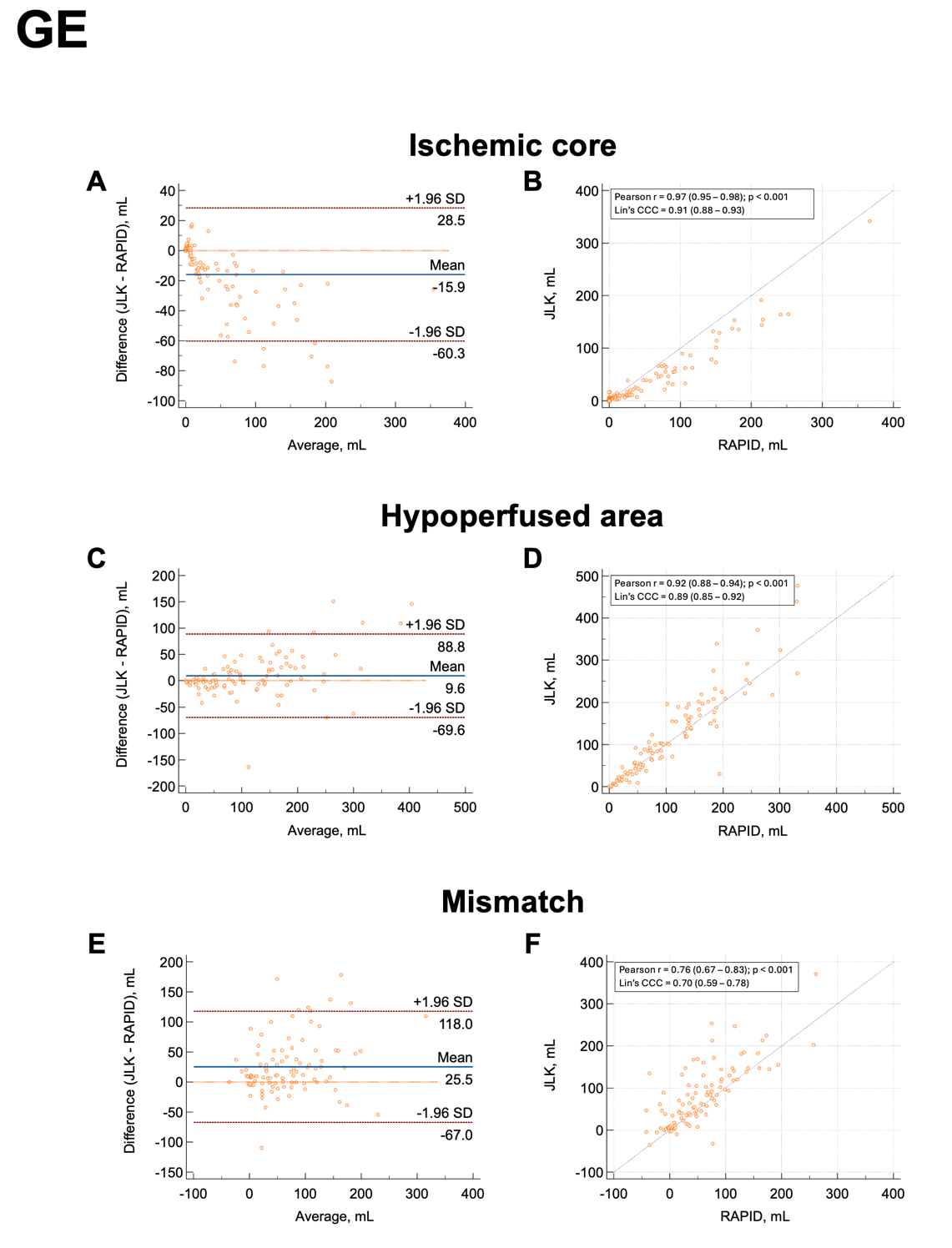
**

**Supplementary Figure 6. Concordance of Ischemic core, Hypoperfused Area, and Mismatch Volumes Between Platforms in patients scanned using Philips MRI systems.**

**
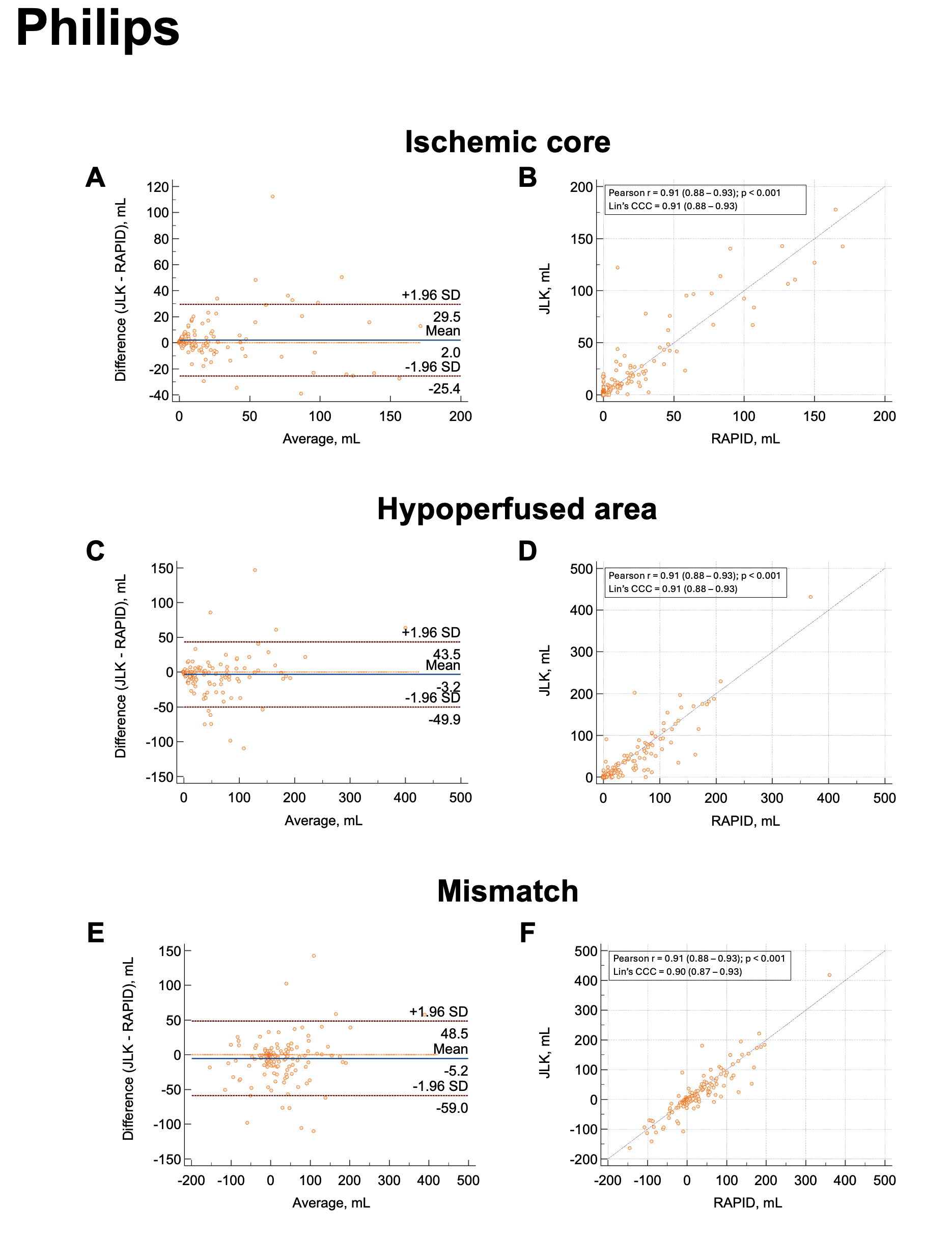
**

**Supplementary Figure 7. Concordance of Ischemic core, Hypoperfused Area, and Mismatch Volumes Between Platforms in patients scanned using Siemens MRI systems.**


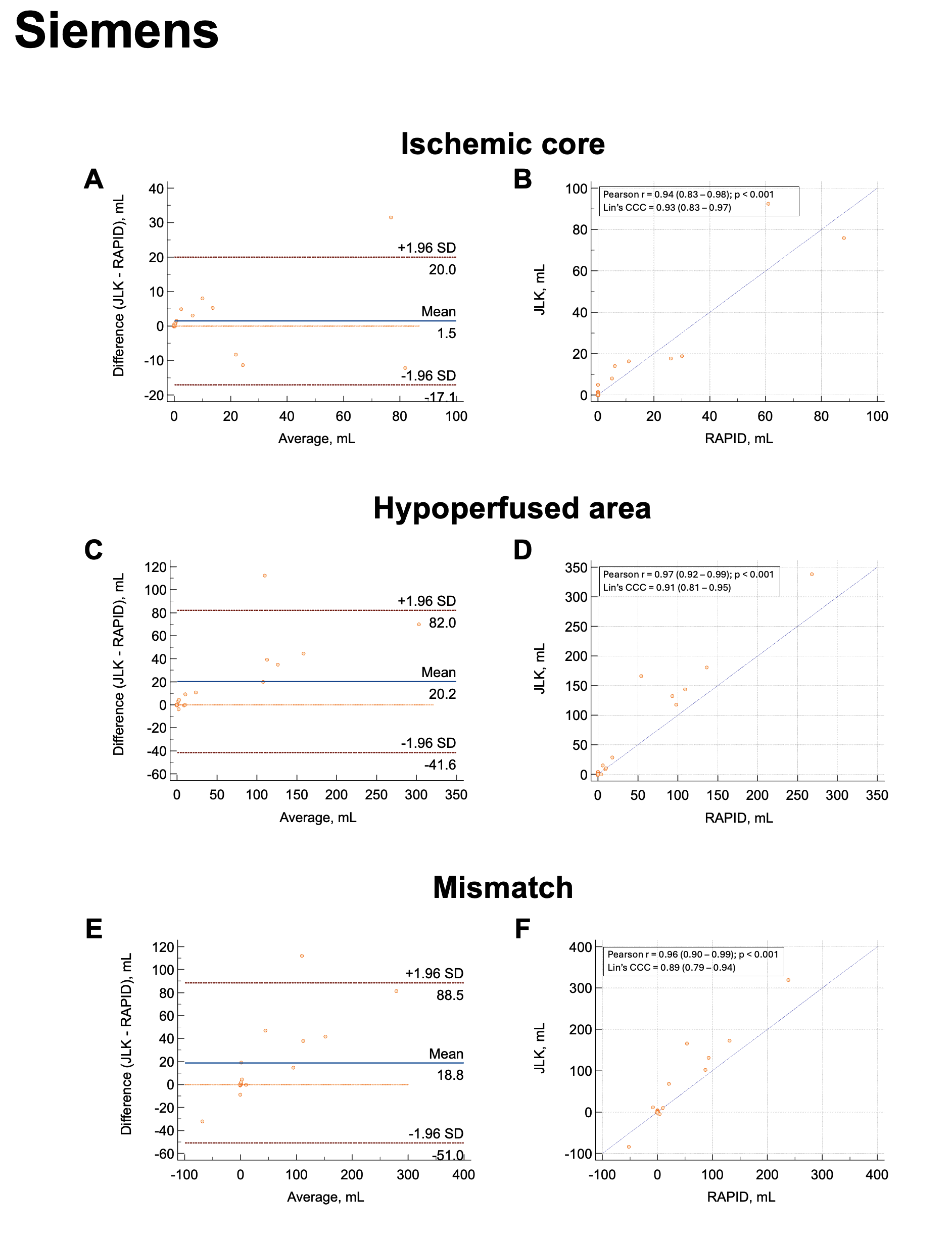

Supplement: Supplementary file 1 [file Data_Sheet_1.DOCX]
